# Resilience in sports through the lens of dynamic network structures

**DOI:** 10.3389/fnetp.2023.1190355

**Published:** 2023-05-19

**Authors:** Yannick Hill, Ruud J. R. Den Hartigh

**Affiliations:** ^1^ Department of Human Movement Sciences, Faculty of Behavioral and Movement Sciences, Vrije Universiteit Amsterdam, Amsterdam Movement Sciences, Amsterdam, Netherlands; ^2^ Institute of Brain and Behaviour Amsterdam, Amsterdam, Netherlands; ^3^ Lyda Hill Institute for Human Resilience, Colorado Springs, CO, United States; ^4^ Department of Psychology, Faculty of Behavioral and Social Sciences, University of Groningen, Groningen, Netherlands

**Keywords:** dynamical systems (DS), growth, mental wellbeing, resistance, performance, physiology, psychology

## 1 Introduction

On and off the sports field, athletes are confronted with numerous stressors. These stressors may reflect daily hassles, heavy training sessions, or occasionally major life events like losing a loved one (Den Hartigh et al., 2022). To prevent injuries or declines in performance and psychological wellbeing, athletes constantly need to demonstrate resilience following these stressors. While some scholars propose that resilience may refer to resisting, recovering from, or growing from a stressor’s negative impact (Masten and Powell, 2003), it has been pointed out that these are distinct concepts and that resilience most closely resembles a recovery-from-stressors process (e.g., Den Hartigh & Hill, 2022; Layne et al., 2008; Layne et al., 2021; Lozano Nasi et al., 2023; Taleb & West, 2023). Therefore, we proceed from the following definition of resilience: “the dynamic process by which a biopsychosocial system returns to the previous level of functioning, following a perturbation caused by a stressor” (Hill et al., 2018b, p. 367).

Defining resilience from such a dynamical perspective in sports implies that resilience emerges from continuously changing interactions between multiple psychological and physiological variables, and that it cannot be reduced to a single set of fixed factors (Hill et al., 2018a; Hill et al., 2018b; Hill et al., 2021). Practically, this means that strategies to successfully deal with a stressor in one situation, may not be effective in another situation. Moreover, changes among the factors do not yield proportional outcomes at the observable level (Kelso, 1995; Nowak & Vallacher, 1998). That is, relatively large changes in various factors may have virtually no impact on whether an athlete can demonstrate resilience, whereas relatively small changes of the same factors close to a tipping point may induce injuries or significant performance and wellbeing declines (cf. Pol et al., 2019). Thus, the way in which different factors dynamically interact and change over time needs to be clarified (Hill et al., 2018a).

In this article, we argue that the dynamic process of resilience in sports provides a logical fit with network structures (Pincus & Metten, 2010). Specifically, we demonstrate how networks a) fit with the contemporary conceptualization of resilience and b) can be studied to provide insights into resilience. Finally, new avenues for future research leveraging network analyses will be provided.

## 2 Understanding resilience through networks

**FIGURE 1:**
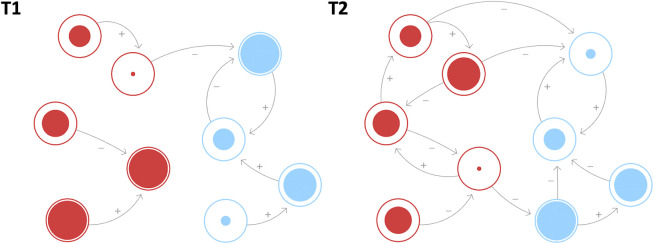
Example of a network with sport-specific psychological (red), and physiological (blue) nodes that changes over time due to intrinsic dynamics. The specific nodes can include motivation and perceived stress on the psychological side as well as training load and muscle tension on the physiological side, but likely differ between individuals (Hill et al., 2021). The nodes change in strength (indicated by their size) and how they are connected to each other. At T1, the individual nodes are relatively loosely connected, but become relatively densely connected across time, which is expressed in the increase in connections as well as the changes in the levels (size) of the variables at T2. As a hypothetical example, due to high motivation, an athlete may invest successively more time and energy into their training, causing the load (and other associated physiological parameters, such as muscular strength) to grow as well. However, once the training load cannot be increased anymore, the connection between motivation and training load may start to dissolve. Instead, the athlete may use their high levels of motivation to focus on mental skills training, leading to the formation of new connections between motivation and arousal management. Note that changes in the nodes and connectivity may also occur following an external perturbation. Whether structural changes in the connectivity are beneficial for an athlete depends on the previous state of the network. Increasing connectivity may be beneficial when the connectivity was too low previously, but problematic when the network becomes too densely connected and therefore rigid (e.g., Scheffer et al., 2012). The figure was created using the free software *Loopy* (https://ncase.me/loopy/).

A network represents a collection of interconnected variables or nodes that exchange information with each other (Balagué et al., 2020; Bartsch et al., 2015; Bashan et al., 2012; Pincus & Metten, 2010). Networks can be used to model small-scaled systems, such as neurons within the brain, as well as large-scaled systems like societies where each node represents a person. In any network, the nodes and their interaction patterns may change over time (see Figure 1; Balagué et al., 2020; Bartsch et al., 2015; Bashan et al., 2012; Den Hartigh and Hill, 2022; Den Hartigh et al., 2018). These so-called *intrinsic dynamics* (Vallacher et al., 2015; Gernigon et al., 2022) allow a network to reorganize itself in response to an external perturbation (Pincus & Metten, 2010; Bashan et al., 2012; Kiefer et al., 2018). In terms of resilience, a network structure may be perturbed by a stressor, but restores its previous configuration over time. Furthermore, due to their dynamic interactions, networks also allow for the nonlinear influences of the constituent nodes (Bashan et al., 2012; Bartsch et al., 2015). That is, the very structure of a network may either amplify or dampen the perturbation of a stressor (Gao et al., 2016). Therefore, network structures and how they change over time in response to perturbations can provide valuable insights into the dynamic process of resilience. Accordingly, recent advances in the domain of physiology have applied network analyses to capture critical transitions on the level of behavior and movement (Kerkman et al., 2020; Garcia-Retortillo and Ivanov, 2022).

### 2.1 Networks that are resilient

In order to prevent injuries or declines in performance and wellbeing in athletes, providing predictions about an athlete’s capacity to demonstrate resilience is an important avenue (Den Hartigh et al., 2022). These predictions may become possible by capturing the structures of relevant networks (Gao et al., 2016). For example, according to Scheffer and colleagues (2012), a network that consists of many tightly coupled nodes may be more prone to collapse following a perturbation compared to a network that shows more heterogeneity in its connectivity. That is, networks with too many nodes characterized by high in-degrees (i.e., a measure of connectivity, Jia and Barabasi, 2013) become increasingly fragile because the perturbation spreads through the entire system causing a “domino effect” (Ghoshal and Barabasi, 2011; Bashan et al., 2012; Scheffer et al., 2012).

The spread of a perturbation throughout a network may further be enhanced or dampened by specific variables that are associated with resilience. For example, protective factors may reduce the perturbation of a stressor and contribute to resistance, while promotive factors may facilitate the reorganization process following a perturbation (Layne et al., 2008; 2021). Simulation studies from the domain of clinical psychology showed that when a risk factor represents a central node with high connectivity, it may enhance the spread of a perturbation throughout a symptom network (Lunansky et al., 2021). These findings have also been verified with empirical data of personality network structures in response to stress (Papageorgiou et al., 2019). In contrast, a central protective factor dampens the perturbation caused by a stressor and avoids the spread from one symptom to another (Kalisch et al., 2019; Papageorgiou et al., 2020). Because several outcomes are relevant for athletes (i.e., physical health, performance, and psychological wellbeing), different protective and risk factors may be specified for different levels of functioning. However, it should be noted that due to the intrinsic dynamics, the role of these nodes may change over time. Therefore, central issues like injury prevention may not only be a question of monitoring the right variables over time (Den Hartigh et al., 2022), but also understanding the changing interconnectivity of these variables (Andrews et al., 2022; Balagué et al., 2022).

### 2.2 Networks that become (less) resilient

Compared to the notion that network structures can explain resilience, the structural changes that networks undergo to become resilient are relatively unexplored (e.g., Pincus & Metten, 2010). In exercise physiology, the general interest in tracking how interactions between organ systems (e.g., brain, heart, skeletal muscles) change in response to fatigue and training through the assessment of “network-based biomarkers” has, however, already gained traction (e.g., Balague et al., 2022). Regarding the concept of resilience, researchers suggested that a system’s underlying structure may change in response to a perturbation to become more rigid and stable or more flexible without losing its functionality (Pincus & Metten, 2010). Specifically, following a perturbation, new connections between individual nodes may be formed (i.e., integration tendency, Kiefer et al., 2018), which allows a system to preserve its stability. In contrast, to avoid becoming trapped in dysfunctional states, connections between specific nodes may be dissolved (i.e., segregation tendency, Kiefer et al., 2018). Thus, while on a superordinate level (i.e., performance or health), we observe a recovery trajectory, the underlying network structure could have undergone changes (Bashan et al., 2012; Bartsch et al., 2015). These changes may help an athlete to bounce back more quickly when similar perturbations occur in the future.^1^ Therefore, the changes in the underlying network structure may explain how resilience in superordinate variables in sports, such as performance or physical and psychological wellbeing, may be improved. For example, when an athlete experiences psychological problems, dissolving tight connectivity between physiological and psychological nodes may be beneficial for an athlete to prevent physical injuries. Conversely, increasing the connectivity may be functional when either psychological or physiological nodes can help buffer against external perturbations (cf. Balagué et al., 2020).

Network structures may also provide insight into resilience losses. Previous research has shown that successive stressors can destabilize a system and reduce the capacity for resilience (Scheffer et al., 2012). In athletes, such resilience losses may be marked by a slowing down in the recovery rate to the previous state (i.e., “critical slowing down”), and can ultimately lead to sudden declines in the athletes’ performance or wellbeing (e.g., Hill et al., 2018a; 2020; Hill et al., 2021; Den Hartigh et al., 2022). This means that the network would require increasingly more time to restore its previous structure.^2^ Mapping the stress-response as well as the time it takes for an athlete to return to the previous state can provide valuable insights into when preventive measures need to be taken (Hill et al., 2018a). Additionally, by gaining a better understanding of the individual-specific configuration of a network, the timed interventions may be targeted at the risk factors at hand (Lunansky et al., 2022). For example, if the recovery in the physical state and wellbeing of an athlete slows down following normal training loads, the training load may be temporarily reduced (e.g., taking a day off) before negative transitions to physical or psychological problems occur.

## 3 Future directions

In order to identify changes in the network structures that underlie resilience, daily measurements of multiple variables would need to be collected. Such data collections may be conducted around the daily training sessions of athletes and can include (but are not limited to) variables like recovery from the previous sessions, motivation to train, training load, or enjoyment of the training session (Den Hartigh et al., 2022). Note that specific analytic strategies for this kind of data are beyond the scope of this paper. Therefore, we refer readers to Pincus and Metten (2010) for different examples of resilience-specific network analyses (see also Blanken et al., 2019), and Hasselman (2022) for a state-of-the-art multiplex recurrence network technique.^3^

Because the above outlined data collection protocols can be time-consuming or unavailable to researchers, alternative approaches may focus on simulation studies. Dynamic network modelling has already been applied to the domain of sports. For example, Den Hartigh and colleagues (2018) used a model based on coupled differential equations to determine talent development in sports. Interestingly, these simulations also contained a perturbation which needed to be overcome in the form of transitioning from the youth to the senior level. Such models can be 1) varied with regards to what variables should be included to represent the nodes and how the interactions between the nodes may change over time, and 2) examined for their specific structure and changes to identify when external stressors cause stronger or weaker perturbations (Gao et al., 2016). Therefore, stimulation studies may be particularly promising when adequate timeseries data collection is limited.

## 4 Discussion

In this article, we discussed the potential of network analyses to provide more in-depth insights into the dynamic process of resilience in sports. We argued that networks yield a striking resemblance with the key properties of resilience and provide a logical fit as a level of analysis. Specifically, the intrinsic dynamics and interaction-dominance of networks may account for nonlinear changes of resilience and explain how the process unfolds over time. We therefore propose that network analyses can provide a powerful future avenue for studying resilience in sports, because they may not only indicate what makes an athlete resilient, but also how resilience in athletes changes over time. From our perspective, the field can make major advances by either closely monitoring and analyzing network structures of physiological and psychological variables of athletes or engaging in simulation studies of how networks respond to perturbations over time.

## Footnotes

1 Note that if a stressor is resisted and no perturbation occurs, the functional reorganization of the network may not occur (Den Hartigh & Hill, 2022).

2 The same functional output may also be achieved from different network configurations (i.e., degeneracy, Edelman & Gally, 2001). Therefore, a precise definition of the level of functioning to which the system returns is warranted.

3 Inspirations for additional sophisticated approaches for network-based analyses can also be inferred from recent publications in the domain of network physiology (e.g., Bashan et al., 2012; Bartsch et al., 2015; Rizzo et al., 2020; Rizzo et al., 2022; Garcia-Retortillo et al., 2023).
